# Cdc6 contributes to abrogating the G1 checkpoint under hypoxic conditions in HPV E7 expressing cells

**DOI:** 10.1038/s41598-017-03060-w

**Published:** 2017-06-07

**Authors:** Hanxiang Chen, Qishu Zhang, Lijun Qiao, Xueli Fan, Weifang Zhang, Weiming Zhao, Jason J. Chen

**Affiliations:** 1 Department of Pathogenic Biology and Key Laboratory of Infection and Immunity of Shandong Province, Shandong University School of Basic Medical Sciences, Jinan, Shandong 250012 China; 2 The Cancer Research Center, Shandong University School of Medicine, Jinan, Shandong 250012 China; 3 Department of Medicine, University of Massachusetts Medical School, Worcester, MA 01532 USA; 4 Department of Dermatology, Xijing Hospital, Fourth Military Medical University, Xian, Shanxi 710032 China

## Abstract

The human papillomavirus (HPV) plays a central role in cervical carcinogenesis and its oncogene E7 is essential in this process. We showed here that E7 abrogated the G1 cell cycle checkpoint under hypoxia and analyzed key cell cycle related proteins for their potential role in this process. To further explore the mechanism by which E7 bypasses hypoxia-induced G1 arrest, we applied a proteomic approach and used mass spectrometry to search for proteins that are differentially expressed in E7 expressing cells under hypoxia. Among differentially expressed proteins identified, Cdc6 is a DNA replication initiation factor and exhibits oncogenic activities when overexpressed. We have recently demonstrated that Cdc6 was required for E7-induced re-replication. Significantly, here we showed that Cdc6 played a role in E7-mediated G1 checkpoint abrogation under hypoxic condition, and the function could possibly be independent from its role in DNA replication initiation. This study uncovered a new function of Cdc6 in regulating cell cycle progression and has important implications in HPV-associated cancers.

## Introduction

Human papillomaviruses (HPVs) are double-strand, non-enveloped small DNA viruses^1^. HPV is one of the most common sexually transmitted infections worldwide^2^. To date, over 170 genotypes of HPV have been identified^3, 4^ and can be classified into two major groups: cutaneous and mucosal HPV. Infection by HPV may lead to the formation of warts, benign lesions, cervical and several other cancers. According to the clinical prognosis of the lesions they cause, mucosal (genital) HPV types can be categorized as either “high-risk” or “low-risk” types. Up to 99% of cervical cancers contain high-risk HPV^5^. In addition, HPV has been detected in over 80% of oropharyngeal cancers^6^.

HPV infects the basal layer of cervical epithelium and then relies on the differentiation of the host cell to complete its life cycle. HPV encodes proteins that promote S-phase re-entry in differentiating keratinocytes^7^. Hence, HPV can manipulate the cell cycle by establishing a milieu in the differentiated keratinocytes supportive for viral DNA amplification. Some of these cell cycle alteration activities may be correlated with HPV-associated carcinogenesis. The E6 oncoprotein leads to the rapid ubiquitination and degradation of p53^8^ while E7 binds and promotes the degradation of pRb, leading to the release of E2F^9^ and uncontrolled cell proliferation^10, 11^. pRb-independent functions of E7 have also been demonstrated^12^. Under normal conditions, DNA damage arrests cells in G1 phase and prevents cells with damaged DNA from multiplying, and allowing the cellular repair systems to fix damaged DNA. E7-expressing cells bypass the G1 arrest induced by DNA damage^13^. The mechanism by which E7 regulates G1 checkpoint has been under extensive study yet is still not fully understood. We have recently shown that Cdk1 and WDHD1 play a key role in G1/S transition in E7-expressing cells^14, 15^.

Cell division cycle 6 (Cdc6) is an essential regulator of DNA replication in eukaryotic cells. The well-established function of Cdc6 is to assemble prereplicative complexes (preRCs) at origins of replication during G1 phase^16^. As a key factor for origin licensing, Cdc6 is responsible for the loading of MCM onto the origins of replication and is essential for the initiation of DNA replication^17^. In G1/S transition, Cdc6 promotes cell cycle progression by activating Cdk2, which is bounded by p21 or p27, in an ATP dependent way^18, 19^. Cdc6 knockdown leads to cell cycle arrest and induces apoptosis^20^. Cdc6 is prone to being overexpressed in most cancer cells because of dysfunction in the pRb-E2F transcriptional pathway^21^. Deregulation of Cdc6 led to the inactivation of the INK4/ARF locus, which encodes three important tumor suppressor genes, p16INK4a, p15INK4b, and p53 activator ARF^22, 23^. Cdc6 has been identified as a biological marker for cervical cancer in early detection^24^. We have recently shown that Cdc6 is up-regulated in E7-expressing cells and plays an important role in E7-mediated re-replication^25^.

The microenvironment of a solid tumor is characterized by irregular vascularization, poor nutrient and oxygen supply. The continuously increasing cell number and the demand of O_2_ exacerbate the hypoxic stress. Hypoxia inducible factor 1α (HIF-1α) is a central molecule involved in mediating these effects in cancer cells. Of note, in general, human cancers express high levels of HIF-1α^26^ not only due to the hypoxic tumor microenvironment, but also because of the dysregulated signaling pathway for catering and adapting the challenging circumstances. As a transcription factor, HIF-1α regulates multiple genes that involved in energy metabolism, angiogenesis^27^ and apoptosis. HIF-1α arrest cell cycle at G1 phase by up-regulating the expression of Cdk inhibitors p21 or p27 under hypoxia^28, 29^. A non-transcriptional mechanism of HIF-1α arrest of cell cycle was also reported^30^. In cervical cancer, HPV E7 increases HIF-1α mediated transcription by inhibiting the binding of histone deacetylases^31^, leading to HIF-1α accumulation and VEGF expression, which may contribute to enhanced angiogenesis^32, 33^. Glioma cells expressing HPV-16 E7 showed a G2/M arrest with concomitant decrease in G1 and S phases subject to hypoxia^34^. The cell cycle profiles in other types of cells expressing HPV E7 under hypoxia remain to be determined.

In this study, we demonstrated that E7 abrogated the hypoxia-induced G1 arrest. We then took a proteomic approach to search for proteins that are differentially expressed in E7 expressing cells under hypoxia. Cdc6 was found to be up-regulated in E7 expressing cells under hypoxia. Significantly, we demonstrated that Cdc6 played a role in E7-mediated G1 checkpoint abrogation under hypoxic condition. This study suggested a new function of Cdc6 in regulating cell cycle progression and has important implications in HPV-associated cancers.

## Materials and Methods

### Cell culture and reagents

Human retinal pigment epithelium cell line (RPE1) was maintained in a 1:1 dilution of DMEM-Ham’s F-12 medium plus 10% FBS. RPE1 cells stably expressing pBabe or HPV-16 E7 were established by retrovirus-mediated infection using the pBabe-puro-based retroviral construct. Cells were selected with 10.5 μg/ml puromycin for 3 to 6 days. After the infected cells were pooled and expanded, they were maintained in puromycin (6.5 μg/ml) and used within 15 passages. The HPV-16 positive human cervical cancer cell line CaSki was purchased from American Type Culture Collection (ATCC). CaSki cells were cultured in DMEM supplemented with 10% FBS and antibiotics.

Deferoxamine (DFO, Sigma) and CoCl_2_ (Sigma) were used to mimic hypoxia at a dose of 200 μM and 500 μM, respectively.

### Cell viability assay

RPE1 vector and E7 cells were seeded in 96-well plates at a density of 7,000 cells per well. After being treated with DFO (from 50 μM to 300 μM) or CoCl_2_ (from 0.1 mM to 1 mM) for 72 hours, the cell viability was determined by the Cell Counting Kit-8 (Dojindo) according to the manufacture and read by the Infinite M200 PRO microplate reader (Tecan Group Ltd.) at 450 nm.

### Flow cytometry

For cell cycle analysis, cells were collected at various time points, fixed with 70% ethanol overnight at 4 °C, resuspended with 50 μg/ml RNaseA plus 50 μg/ml propidium iodide (PI), and then analyzed by fluorescence-activated cell sorting (FACS, Becton Dickinson). Cell cycle data analysis was done using Modfit LT (version 3.10). Each histogram is consisted of the Data Line, Model Fit Line and the model components. The beginning of the S-phase Rectangle is the mean position of the G1 Gaussian and the end is at the mean position of the G2/M Gaussian^35, 36^.

For the bromodeoxyuridine (BrdU) labeling experiment, we used a FITC BrdU Flow Kit (BD Biosciences, San Diego, CA) and followed the manufacturer’s instruction. The BrdU data was analyzed by using FCS Express.

### Immunoblotting

Total cell lysates were prepared in RIPA lysis buffer (Santa Cruz). The protein concentration was measured by the bicinchoninic acid (BCA) protein assay reagent (Invitrogen). Equal amounts of protein from each cell lysate were separated in an SDS polyacrylamide gel (PAGE) and transferred onto a polyvinylidene difluoride (PVDF) membrane. Membranes were reacted with antibodies against pRb (BD Biosciences), HIF-1α (BD Biosciences), Cdc6 (Santa Cruz), Cdk1 (Santa Cruz), Cdk2 (Santa Cruz), p53 (BD Biosciences), p21 (BD Biosciences) and p27 (Santa Cruz). HRP conjugated goat anti-mouse or anti-rabbit were used as secondary antibodies. GAPDH (Santa Cruz) or β-tubulin (Sigma) was used to indicate loading amount of total proteins. ImageJ (NIH) was used to quantify gel images.

### RNA interference (RNAi)

Cells were seeded onto a 60 mm dish the day before transfection. Cells were transfected with a final concentration of 20 nM siRNA per target gene using Lipofectamine 2000 transfection reagents according to the manufacturer (Invitrogen). For gene knockdown analysis, cells were harvested 72 hours post-transfection and specific protein levels were analyzed by immunoblotting. For cell cycle analysis, twenty-four hours after transfection, cells were treated with DFO (200 μM) for an additional 8 hours. All of the siRNAs were synthesized from Sigma and the oligo-nucleotide sequences were as follows: siCdc6 sense strand: 5′-CUUCCCACCUUAUACCAGAdTdT-3′; siRNA209 (siE6) sense strand: 5′-UCCAUAUGCUGUAUGUGAUdTdT-3′; siRNA198 (siE6E7) sense strand: 5′-GCACACACGUAGACAUUCGdTdT-3′. Negative control siRNA (siCon) sense strand: 5′-UUCUCCGAACGUGUCACGUdTdT-3′.

### Reverse-transcription PCR and real-time PCR assay

Total RNA was isolated using TRIzol reagent (Invitrogen) and then transcribed to cDNA using PrimeScriptTM RT Reagent KIT with gDNA Eraser (TaKaRa). Real-time quantitative PCR was performed by the Bio-Rad CFX96 Touch Real-Time PCR Detection system. The levels of mRNA were measured by SYBR Green assay using SYBR® Premix Ex TaqTM (TaKaRa) according to the manufacturer’s instructions and GAPDH was used as a control housekeeping gene. Melting curve analysis was performed to confirm amplification of specific transcripts. Each reaction was run parallel and in triplicate. The expression levels of transcripts were calculated by the relative quantification (2−ΔΔCt) study method. All primer sequences were listed as follows: Cdc6 (forward, 5′-ACCTATGCAACACTCCCCATT-3′; reverse, 5′-TGGCTAGTTCTCTTTTGCTAGGA-3′), HIF-1α (forward, 5′-CACCACAGGACAGTACAGGAT-3′; reverse, 5′-CGTGCTGAATAATACCACTCACA-3′), Cdk1 (forward, 5′-AAACTACAGGTCAAGTGGTAGCC-3′; reverse, 5′-TCCTGCATAAGCACATCCTGA-3′), Cdk2 (forward, 5′-CCAGGAGTTACTTCTATGCCTGA-3′; reverse, 5′-TTCATCCAGGGGAGGTACAAC-3′), GAPDH (forward, 5′-GCACCGTCAAGGCTGAGAAC-3′; reverse, 5′-TGGTGAAGACGCCAGTGGA-3′), HPV-16 E6 (forward, 5′-ACAAACCGTTGTGTGATTTGTT-3′; reverse, 5′-CAGTGGCTTTTGACAGTTAATACA-3′), HPV-16 E7 (forward, 5′-GAACCGGACAGAGCCCATTA-3′; reverse, 5′-ACACTTGCAACAAAAGGTTACA-3′).

### Mass spectrometry

Sample preparation was performed according to filter aided sample preparation (FASP) method^37^. The prepared digests were analyzed by LC-MS/MS using a Thermo EASY-nLC II system (Thermo Scientific, Denmark), which was interfaced to an LTQ Orbitrap Elite/Velos Pro mass spectrometer (Thermo Scientific). LC MS/MS files were exported from XCalibur. Protein identification was subjected to strict data QC filtering within MASCOT and SQUEST. Label-free quantification of identified proteins was performed by Progenesis LC-MS software package from Nonlinear Dynamics.

Functional classification and pathway analysis of differentially expressed proteins (cut-off point 1.75) were analyzed by gene ontology (GO) via DAVID Bioinformatics Resources 6.7. For GO analyses, at least two genes were needed to be present in a cluster, and a probability (*P*) value below 0.05 was considered significant.

### Statistical analysis

All data are shown as means and standard error of the mean (SEM). The Student t test was used to compare the differences between means. Significance was set at a *P* value of < 0.05.

## Results

### HPV-16 E7 abrogates G1 checkpoint under hypoxia in epithelial cells

**Figure 1:**
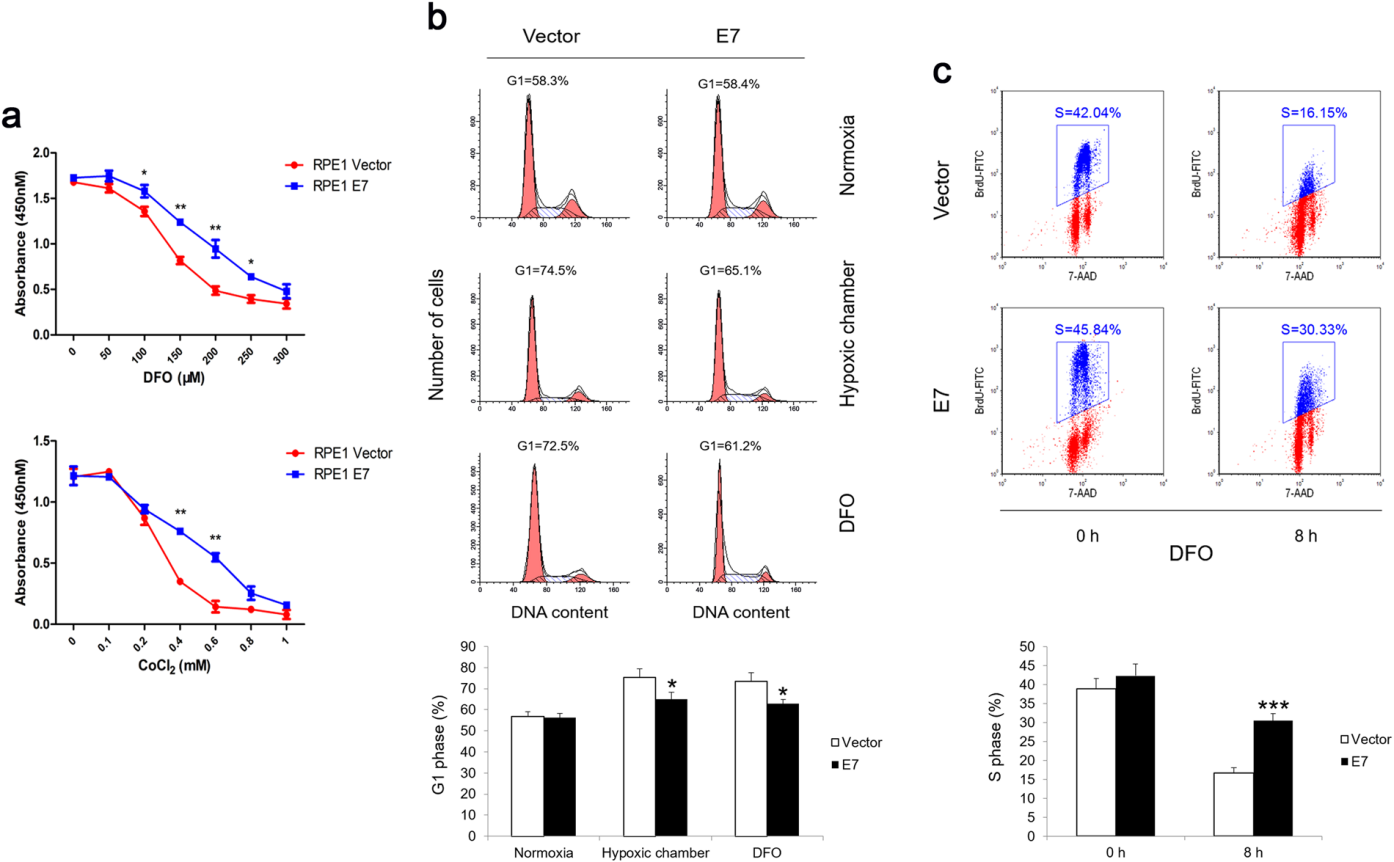
HPV-16 E7 abrogates hypoxia-induced G1 arrest. (**a**) RPE1 E7 cells were treated with hypoxia mimic drug DFO or CoCl_2_ at different concentrations for 72 hours, cell viability was determined using CCK8 assay. Data from a representative of 3 experiments are shown. (**b**) RPE1 vector and RPE1 E7 cells were incubated in hypoxic chamber at 1% oxygen or with 200 μM DFO for 8 hours, respectively. Cells were stained by PI and examined by flow cytometry. Data from a representative of 4 experiments are shown (Upper panel) and are summarized (Lower panel). (**c**) RPE1 vector and E7 expressing cells were treated with 200 μM DFO for 6 hours and labeled with 20 nM BrdU for 2 additional hours. Cells were stained with anti-BrdU antibody, counterstained with 7-AAD, analyzed by flow cytometry. Data from a representative of 4 experiments are shown (Upper panel) and the percentage of BrdU-positive cells was gated. Data are summarized (Lower panel). Error bars reflected the standard error of the mean. *P < 0.05; **P < 0.01; ***P < 0.001.

Hypoxia has been shown to inhibit cell proliferation in a wide variety of cell types while cancer cells could proliferate to some extent under hypoxic condition^38–41^. Here we assessed the effect of HPV-16 E7 on cell cycle progression under hypoxia. RPE1 cells stably expressing HPV-16 E7 were established^42^. Cell viability was analyzed after treating cells with DFO or CoCl_2_ to mimic hypoxic conditions. As shown in Fig. 1a, the cell viability of control cells was attenuated with increasing drug concentration while E7 expressing cells had a significantly higher rate. To examine cell cycle profiles of E7 expressing cells under hypoxia, cells were cultured in hypoxic chamber at 1% O_2_, or treated with DFO and then analyzed by flow cytometry. In hypoxic chamber, 74.5% of vector cells accumulated in G1 phase while less (65.1%) E7 expressing cells arrested at G1 (Fig. 1b). Similar observations were found after DFO treatment (72.5% vector cells and 61.2% E7 expressing cells at G1) (Fig. 1b), suggesting that hypoxia induced cell cycle arrest in G1 phase was partly abrogated by HPV-16 E7. To examine the effect of E7 in promoting S-phase entry of cells under hypoxia more directly, we measured BrdU incorporation after treating E7 expressing cells with DFO. Significantly, much more BrdU incorporation (30.3% vs. 16.1%) was observed in RPE1 E7 cells compared with vector control cells (Fig. 1c). Similar results were observed when cells were treated with CoCl_2_ (data not shown). These results demonstrate that E7 facilitates S-phase entry under hypoxia.

### Hypoxia leads to G1 arrest in HPV-16 E7 knockdown cervical cancer cells

**Figure 2:**
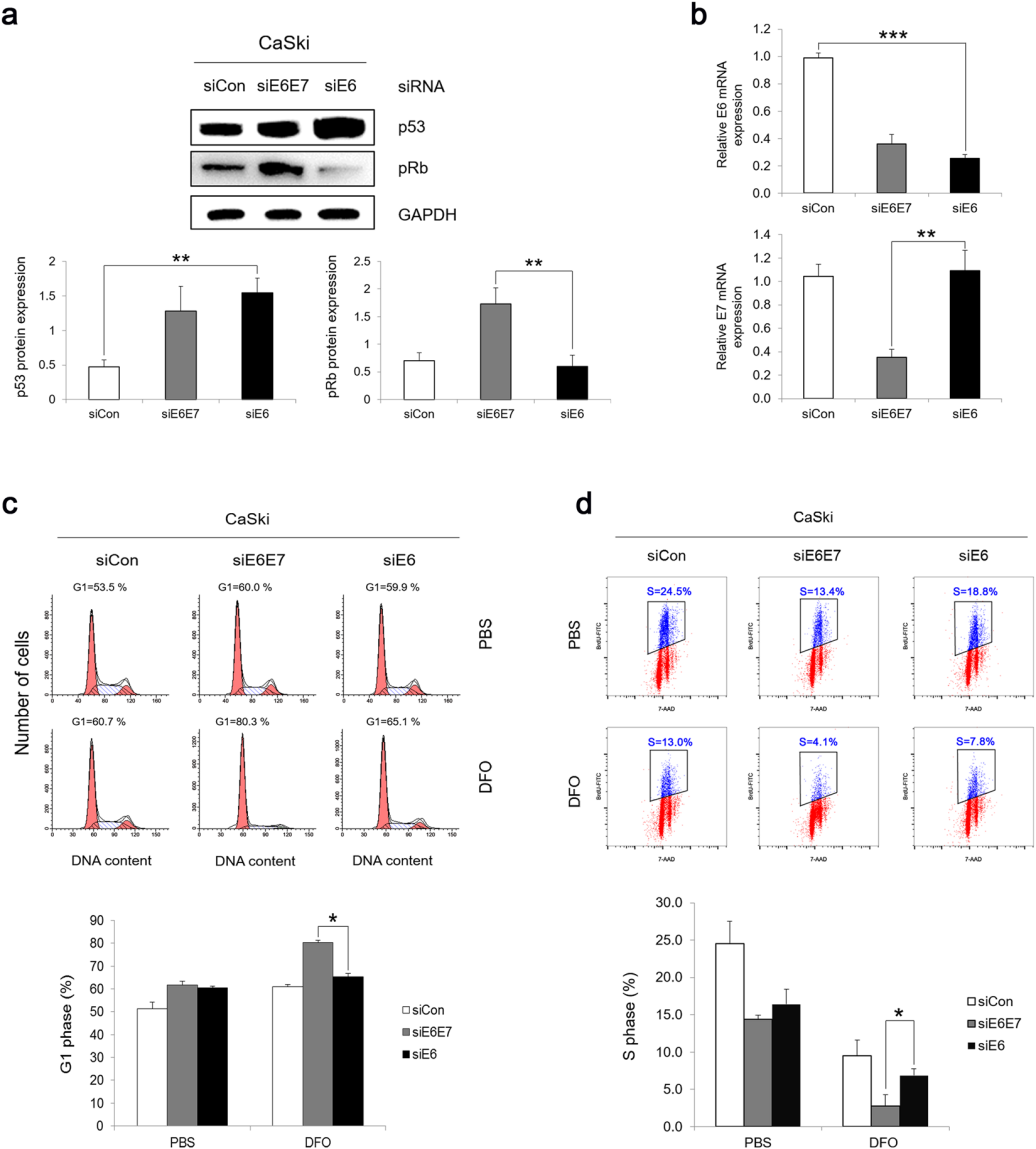
Hypoxia leads to G1 arrest in HPV-16 E7 knockdown cervical cancer cells. HPV-16 positive CaSki cells were transfected with siRNAs targeting E7 or E6 (siRNA198 or siRNA209). (**a**) Forty-eight hours later, the steady-state levels of p53 and pRb were examined by Immunoblotting (full-length blots are presented in Supplementary Figure 1). (**b**) Thirty-six hours post-transfection, the mRNA levels of HPV-16 E6 and HPV-16 E7 were determined by RT-qPCR. (**c**) Twenty-four hours post-transfection, cells were treated with 200 μM DFO for 8 hours. Cells were stained with PI and analyzed by flow cytometry. A representative experiment of 3 is shown (Upper panel) and summarized (Lower panel). (**d**) Twenty-four hours after transfection, cells were treated with 200 μM DFO for 6 hours and then labeled with BrdU for 2 additional hours. Cells were stained with anti-BrdU antibody, counterstained with 7-AAD, analyzed by flow cytometry. Data from a representative of 3 experiments are shown (Upper panel) and the percentage of BrdU-positive cells was gated. Mean percentage of BrdU positive CaSki cells are summarized (Lower panel). Error bars reflected the standard error of the mean. *P < 0.05; **P < 0.01; ***P < 0.001.

Given the association between HPV-16 E7 and cell cycle progression under hypoxia, we examined the effect by using RNAi technology in HPV-16 positive cervical carcinoma cells. CaSki cells are derived from a cervical cancer patient and contain around 600 integrated HPV-16 genome copies. In high-risk HPV associated cervical cancer cells, E6*I is an extremely efficient spliced transcript and the mRNAs of E6*I are responsible for HPV-16 E7 production^43^. The siRNA targeting E7 potentially impact the expression of E6. Given by this, in order to examine the function of E7 on hypoxia-induced G1 arrest, we utilized siRNA198 and siRNA209 to selectively knockdown HPV-16 E6E7 and E6, respectively^44^. The expressions of E6 and E7 targets, p53 and pRb, were used to evaluate the knockdown efficiency. As shown in Fig. 2a, after E6 knocked down, the expression of p53 was increased in both CaSki-siE6E7 and CaSki-siE6 cells. Compared with siE6, the expression of pRb was increased in CaSki-siE6E7 cells, indicating the knockdown of E7. We also assessed the expression of E6 and E7 at mRNA level (Fig. 2b). Knockdown E6E7 decreased both E6 and E7 mRNA expressions while knockdown E6 specifically impaired the expression of E6. These results suggest that CaSki-siE6E7 cells could be used as control for CaSki-siE6 cells in studying the function of E7. Notably, E6 knockdown led to increased p53 protein expression, which is consistent with previous observations of p53 upregulation in E7 expressing cells^45^.

We next determined the cell cycle profiles of CaSki-siE6E7 and CaSki-siE6 cells under hypoxia. Both E6E7 and E6 knockdown in CaSki cells led to an increased number of cells at the G1 phase (Fig. 2c). After being treated with DFO, 65.1% of CaSki-siE6 cells stayed in G1 phase while 80.3% of CaSki-siE6E7 cells accumulated at G1 (Fig. 2c), indicating that E7 promotes G1/S transition under hypoxia in cervical cancer cells.

To demonstrate the role of E7 in promoting S-phase entry of cervical cancer cells more directly, we measured BrdU incorporation upon DFO treatment. We found that knockdown of E7 by siRNAs led to a significant reduction in BrdU incorporation (from 7.8% for CaSki-siE6 to 4.1% for CaSki-siE6E7) after DFO treatment (Fig. 2d). These results demonstrate an important role of E7 in the G1 cell cycle control and S-phase entry of cervical cancer cells under hypoxia.

### Level of cell cycle-related proteins in E7 expressing cells under hypoxic condition

**Figure 3:**
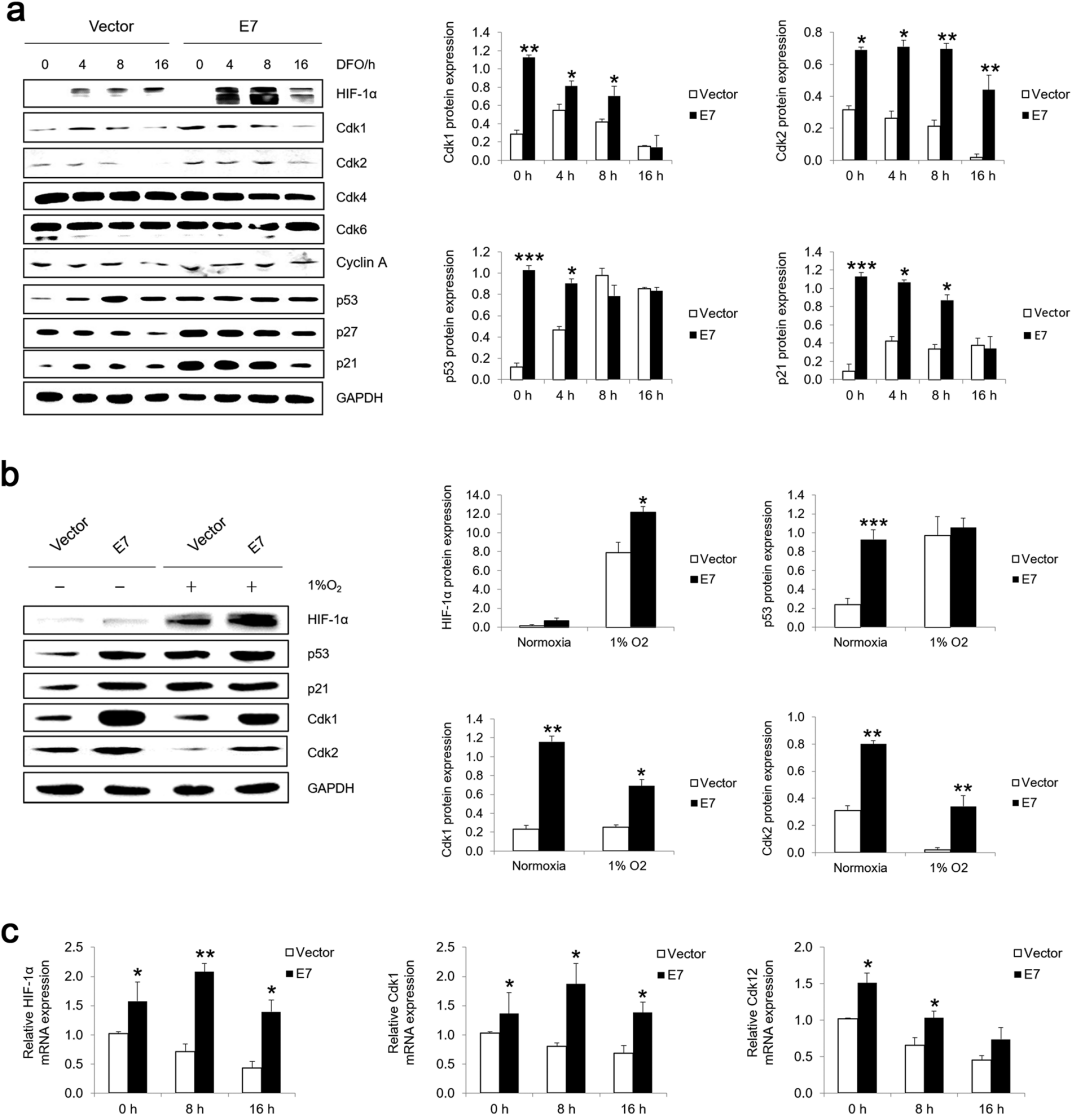
Expression of cell cycle-related proteins under hypoxic conditions. The steady-state levels of HIF-1α, p53, p21, p27, Cdk1, Cdk2, Cdk4, Cdk6 and cyclinA in RPE1 cells treated with 200 μM DFO (**a**) or 1% O_2_ chamber (**b**) were examined by immunoblotting (Left panels) (original blots are presented in Supplementary Figure 2) and quantified (Right panels). A representative of 3 independent experiments is shown. (**c**) The mRNA level of HIF-1α, Cdk1 and Cdk2 were detected by RT-qPCR. GAPDH was used as a control. *P < 0.05; **P < 0.01; ***P < 0.001.

To understand the mechanism by which HPV-16 E7 abrogates hypoxia-induced G1 arrest, we examined the expressions of cell cycle-related proteins under hypoxic condition. As shown in Fig. 3a, the steady-state level of hypoxia marker HIF-1α was higher in E7 expressing cells compared with the vector control cells and increased after DFO treatment. These results are consistent with a previous report^31^. Both Cdk1 and Cdk2 levels were decreased under hypoxia while their expression levels were still higher in E7 expressing cells until 8 hours post treatment. No significant differences were found for the expressions of Cdk4, Cdk6 and cyclin A after DFO treatment. The level of p53 remained high in DFO-treated E7 expressing cells. In contrast, although p53 expression was low in the vector control cells, it was elevated after DFO treatment. Both p27 and p21 expressions showed similar patterns until the treatment time reached 16 hours, where they dropped (Fig. 3a). We also examined the expressions of a few selected cell cycle related proteins after cells were cultured in low oxygen chamber. As shown in Fig. 3b, the steady-state level of HIF-1α was higher in E7 expressing cells and increased after cells were cultured with 1% O_2_ for 8 hours. The expressions of p53 and p21 did not change much in E7 expressing cells but increased in vector cells after culturing in low oxygen. Both Cdk1 and Cdk2 expressions were decreased after cells were cultured in hypoxic chamber.

Next we determined the mRNA levels of HIF-1α, Cdk1 and Cdk2. As shown in Fig. 3c, compared with the vector control cells, the level of HIF-1α was higher in regularly cultured E7 expressing cells. Upon DFO treatment, the level of HIF-1α mRNA in E7 expressing cells went up at 8 hours post DFO treatment and dropped at 16 hours post treatment. In the vector control cells, however, the level of HIF-1α mRNA decreased after DFO treatment. A similar pattern of mRNA expression was observed for Cdk2 in both vector and E7 expressing cells (Fig. 3c). The level of Cdk1 mRNA was also decreased after DFO treatment. Nonetheless, it was consistently higher in E7 expressing cells than the vector control cells. In summary, under hypoxic condition, the mRNA levels of HIF-1α, Cdk1 and Cdk2 were consistently higher in E7 expressing cells, suggesting that Cdk1 and Cdk2 may counteract the negative effect of HIF-1α on cell cycle.

### Proteomic analysis of E7 abrogation of G1 checkpoint under hypoxia

**Figure 4:**
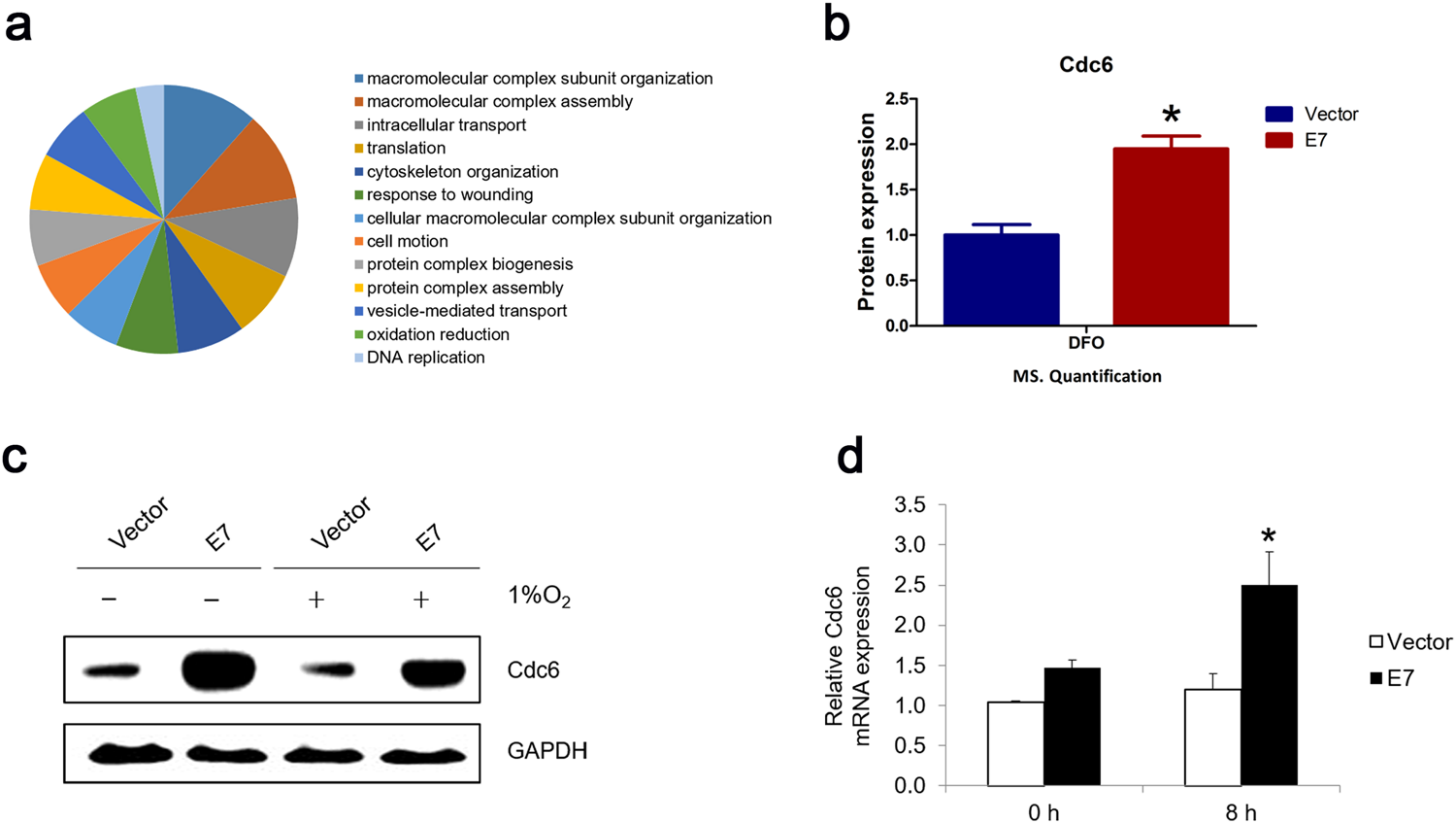
Label-free mass spectrometry quantification of RPE1 vector and E7 expressing cells under hypoxia. (**a**) RPE1 vector and E7 expressing cells treated with 200 μM DFO were prepared for mass spectrometry. Functional classification of differentially expressed proteins that were quantified by M.S. (**b**) Expression level of Cdc6 in vector and E7 expressing cells under hypoxia was determined by M.S. (**c**) Verification of Cdc6 steady-state level in vector and E7 expressing cells treated in hypoxic chamber at 1% oxygen by immunoblotting (original blots are presented in Supplementary Figure 3). (**d**) Verification of Cdc6 mRNA level in vector and E7 expressing cells treated with 200 μM DFO by RT-qPCR. Error bars reflected the standard error of the mean. *P < 0.05.

To systematically explore the mechanism by which HPV-16 E7 abrogates hypoxia-induced G1 arrest, we took a proteomic approach and performed label-free mass spectrometry to search for proteins that are differentially expressed between E7 expressing cells and vector control cells under hypoxia. One hundred and eighty-two differentially expressed proteins were identified at cut-off point 1.75 fold (Supplementary Table 1). Among these proteins, many of them ﻿are implicated in E7 associated carcinogenesis, such as PAK2^46^ and SPARC^47^. We then conducted functional classification and pathway analysis. Pathways with altered protein member expression include translation, cell motion, protein complex assembly, DNA replication and oxidation reduction (Fig. 4a). These pathways are consistent with known biological activities of E7, which has been reported to regulate multiple genes to modulate translation^48^, promote cell migration^49^, play a role in macromolecular complex subunit organization^50^, DNA replication^51^ and redox balancing^52^.

Among the differentially expressed proteins, Cdc6 draws our attention. We have recently demonstrated that Cdc6 is upregulated in HPV E7 expressing cells and plays an important role in E7-induced re-replication^25^. In addition, it has been shown that Cdc6 activates p21 or p27 bound Cdk2-cyclin A/E complexes that may potentially contribute to cell cycle progression. Our M.S. study showed that the protein level of Cdc6 in E7 expressing cells was 1.79-fold higher than in the vector control cells under hypoxic condition (Fig. 4b). We then confirmed the protein expression of Cdc6 by Immunoblotting after cells were treated in hypoxic chamber (Fig. 4c). In normoxia, consistent with what we observed^25^, Cdc6 expression was higher in E7 expressing cells. Under hypoxia, Cdc6 protein level decreased in both vector and E7 expressing cells. However, the steady-state level of Cdc6 was still higher in E7 expressing cells. The mRNA level of Cdc6 was also higher in E7 expressing cells (Fig. 4d). Interestingly, in response to hypoxia, the level of Cdc6 mRNA was increased and higher in E7 expressing cells, suggesting that the Cdc6 protein was degraded more rapidly in E7 expressing cells.

### Cdc6 is important for E7 cells to bypass G1 checkpoint under hypoxia

**Figure 5:**
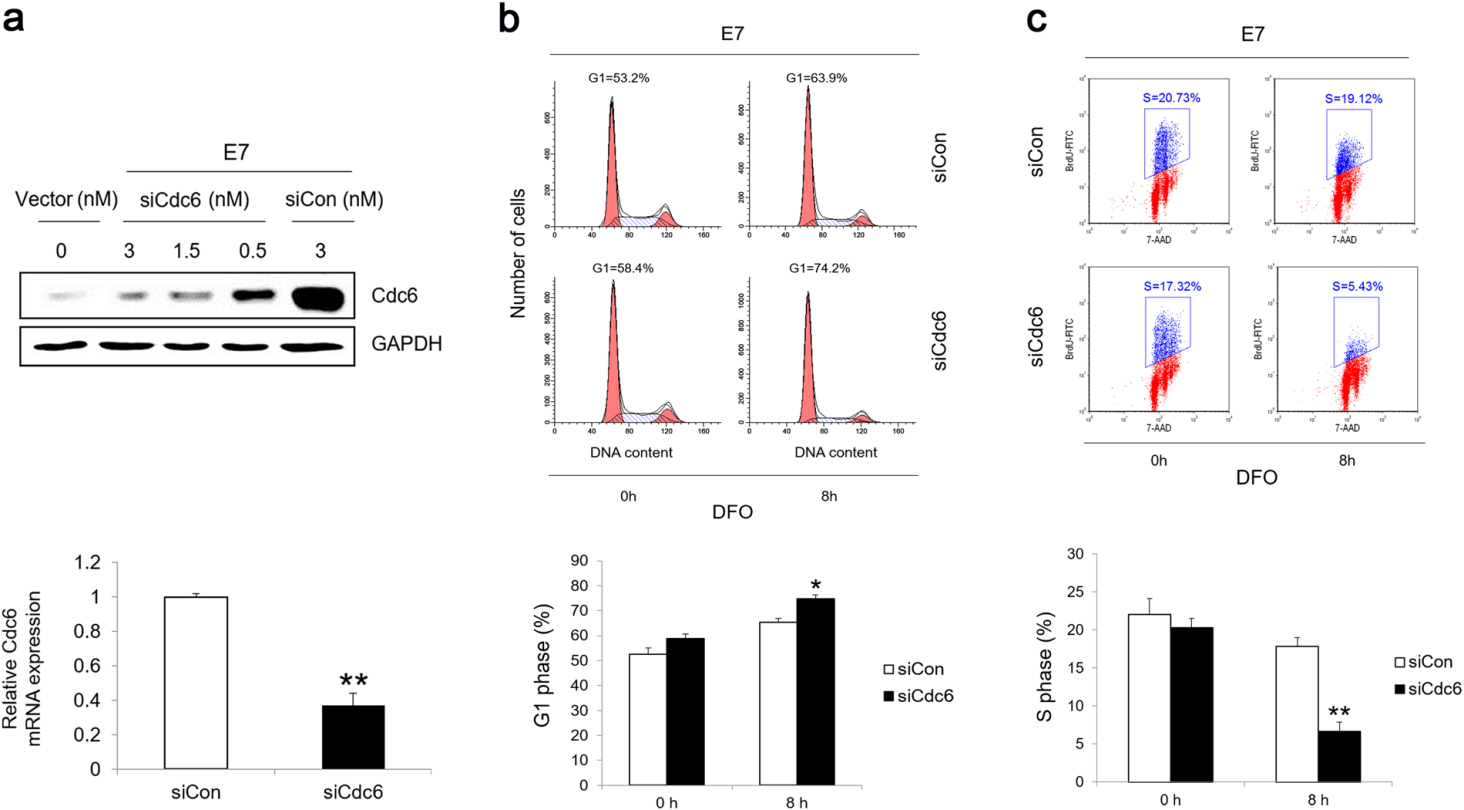
Cdc6 is important for E7 expressing cells to alleviate G1 arrest under hypoxia. (**a**) Cdc6 in RPE1 E7 cells was knocked down by siRNA at indicated doses and analyzed by immunoblotting (Upper panel) (original blots are presented in Supplementary Figure 3) and RT-qPCR (Lower panel, 3 nM siRNA was used). (**b**) RPE1 E7 cells were transfected with either Cdc6 or negative control siRNA. Cells were treated with 200 μM DFO 24 hours post-transfection and cultured for an additional 8 hours, stained with PI and analyzed by flow cytometry. A representative experiment of 3 is shown (Upper panel) and summarized (Lower panel). (**c**) RPE1 E7 cells were transfected with either Cdc6 or negative control siRNA. Twenty-four hours later, cells were treated with 200 μM DFO for 6 hours and then labeled with BrdU for 2 additional hours. Cells were stained with anti-BrdU antibody, counterstained with 7-AAD, analyzed by flow cytometry. Data from a representative of 3 experiments are shown (Upper panel). Data are summarized (Lower panel). Error bars reflected the standard error of the mean. *P < 0.05; **P < 0.01.

Since we have observed that Cdc6 was one of the noticeable genes up-regulated in response to hypoxia, we decided to examine its contribution to E7-mediated G1 checkpoint abrogation in response to hypoxia. We took an RNAi approach to knockdown Cdc6 in the cells. At a final concentration of 3 nM siRNA, Cdc6 was reduced to a level close to what was observed in the vector control cells (Fig. 5a). Under such a condition, the normal DNA replication function of Cdc6 should be intact.

Accordingly, we knocked down Cdc6 and the cell cycle profile of E7 expressing cells under hypoxia was examined. Notably, the effect of HPV 16 E7 on abrogating G1 checkpoint under hypoxia was abolished after knocking down Cdc6. Significantly, more cells were at G1 after Cdc6 knockdown (63.9% for negative control siRNA while 74.2% for Cdc6 siRNA) (Fig. 5b). Next we performed BrdU assay to assess S phase entry in E7 expressing cells after Cdc6 was knocked down. As shown in Fig. 5c, the number of cells in S phase was reduced from 19.1% to 5.4% after Cdc6 knockdown. These results indicate that Cdc6 is critical for E7 expressing cells in bypassing G1 checkpoint under hypoxic conditions.

## Discussion

In this study, we showed that HIF-1α was up-regulated in HPV-16 E7 expressing cells. While hypoxia results in cell cycle arrest of normal cells at G1 phase of the cell cycle via HIF-1α, HPV-16 E7 expressing cells bypassed this G1 checkpoint. To explore the mechanism by which E7 abrogates hypoxia-induced G1 arrest, we used mass spectrometry and identified nearly 200 proteins that are differentially expressed in E7 expressing cells under hypoxia. Among the proteins identified, Cdc6 has been implicated in G1 checkpoint regulation and exhibits oncogenic activities. Our studies demonstrated that Cdc6 was required for E7-mediated G1 checkpoint abrogation under hypoxic conditions. This study uncovered a new function of Cdc6 that might be involved in regulating cell cycle progression under hypoxia and has important implications in HPV-associated cancers.

One important characteristic of solid tumor is poor oxygen supply. Hypoxia leads to increased production of HIF-1α that may lead to angiogenesis^27^ and reduced cell proliferation. In cervical cancer, the high expression of HIF-1α leads to increased VEGF expression and angiogenesis^31–33^. The cell cycle profiles in cells expressing HPV E7 under hypoxia were not clear. Glioma cells expressing HPV-16 E7 showed a concomitant decrease in G1 and S phases subject to hypoxia^34^. Here we demonstrated that HPV-16 E7 abrogates G1 checkpoint under hypoxia and rendered cells enter into S-phase. This function of E7 may contribute to cervical carcinogenesis.

In this study, we uncovered a new function of Cdc6 controlling G1 checkpoint under hypoxic conditions. We believe the elevated Cdc6 in E7 expressing cells was resulted from the deregulation of the pRb-E2F transcriptional pathway. Despite elevated levels of p53, p21 and p27 were found in E7 expressing cells, they were considered dysfunctional^53, 54^. It is believed that DNA replication and cell cycle progression are coupled^55^, we speculated that the DNA replication licensing factors may play a role in cell cycle checkpoint control^56^ indirectly. Therefore, the observed G1 arrest after Cdc6 knockdown could be a result of DNA replication initiation issues instead of a G1 checkpoint response. However, the level of Cdc6 after siRNA knockdown was comparable to that of the vector control cells, suggesting that the regular DNA replication process was intact. These results suggest that G1 checkpoint regulation and DNA replication initiation are separate functions of Cdc6.

Although our study demonstrated a role for Cdc6 in bypassing G1 checkpoint in E7 expressing cells, the mechanism remains to be established. Previous studies have demonstrated that Cdc6 activates p21-associated Cdk2 in an ATP-dependent way^18^. Since we have reported that Cdk1 plays a critical role in DNA damage-induced G1 checkpoint abrogation by E7, we speculate that Cdc6 may work through Cdk1 in bypassing the G1 checkpoint. Future study should determine whether Cdk1 indeed contributes to G1 checkpoint abrogation in E7 expressing cells under hypoxia.

In summary, HPV-16 E7 contributed to G1 checkpoint abrogation under hypoxia by up-regulating Cdc6. We demonstrated Cdc6 was a key regulator in G1/S transition, which may modulate the interaction between p21 and Cdk1. These results reveal that a new function of Cdc6 might be involved in promoting cell cycle progression under hypoxic condition in HPV-16 E7 expressing cells, and may have important implications in HPV-associated cancers.

## Electronic supplementary material

Supplementary Information
