# Supplementary material for: Cdc6 contributes to abrogating the G1 checkpoint under hypoxic conditions in HPV E7 expressing cells
Source: Sci Rep. 2017 Jun 7;7:2927. doi: 10.1038/s41598-017-03060-w (PMC5462782; doi:10.1038/s41598-017-03060-w)

Supplementary Information

Cdc6 contributes to abrogating the G1 checkpoint under hypoxic conditions in HPV E7 expressing cells

Hanxiang Chen[1], Qishu Zhang[2], Lijun Qiao[2], Xueli Fan[3,4], Weifang Zhang[1], Weiming Zhao[1,*] and Jason J. Chen[2,*]

[1]Department of Pathogenic Biology and Key Laboratory of Infection and Immunity of Shandong Province, Shandong University School of Basic Medical Sciences, Jinan, Shandong 250012, China

[2]The Cancer Research Center, Shandong University School of Medicine, Jinan, Shandong 250012, China.

[3]Department of Medicine, University of Massachusetts Medical School, Worcester, MA 01532, USA.

[4]Department of Dermatology, Xijing Hospital, Fourth Military Medical University, Xian, Shanxi 710032, China.

[*]Corresponding authors: Jason J. Chen, Email: jxchen@sdu.edu.cn. Weiming Zhao, Email: zhaowm@sdu.edu.cn.

Supplementary Table 1:

Proteins up-regulated in RPE1 E7 expressing cells

Accession	Score	Fold	UniProtKB ID
P46821|MAP1B	155.47	∞	P46821
P42167|LAP2B	77.73	∞	P42167
Q15102|PA1B3	66.12	∞	Q15102
B4DJ66|B4DJ66	166.13	186.96	B4DJ66
P61970|NUTF2	21.53	151.69	P61970
B4DH52|B4DH52	47.06	73.21	B4DH52
Q00059|TFAM	24.95	56.1	Q00059
P50552|VASP	61.73	23.63	P50552
Q03405-2|UPAR	46.17	18.81	Q03405-2
F5GYX3|F5GYX3	48.16	13.61	F5GYX3
F5H6I5|F5H6I5	120.06	11.44	F5H6I5
P46977|STT3A	43.8	11.4	P46977
Q96C19|EFHD2	77.71	10.25	Q96C19
Q53F19-2|CQ085	26.03	8.6	Q53F19-2
Q9Y696|CLIC4	146.58	8.47	Q9Y696
Q92973-2|TNPO1	45.47	7.65	Q92973-2
O96019-2|ACL6A	41.29	7.23	O96019-2
Q9H3M7|TXNIP	39.05	6.69	Q9H3M7
O00186|STXB3	42.88	6.56	O00186
B7Z5D9|B7Z5D9	50.62	5.68	B7Z5D9
O15372|EIF3H	143.42	4.97	O15372
P50213-2|IDH3A	46	4.91	P50213-2
F5GXL2|F5GXL2	72	4.68	F5GXL2
Q8TF72|SHRM3	59.8	4.66	Q8TF72
C9JYY7|C9JYY7	32.33	4.48	C9JYY7
Q8WW12-2|PCNP	36.62	4.47	Q8WW12-2
A6NN80|A6NN80	455.74	4.37	A6NN80
B4DHC4|B4DHC4	186.28	3.96	B4DHC4
B7Z1R5|B7Z1R5	68.6	3.86	B7Z1R5
P07814|SYEP	184.38	3.75	P07814
A8MZ62|A8MZ62	32.17	3.28	A8MZ62
F5H6X6|F5H6X6	563.51	3.2	F5H6X6
Q9Y4D7-2|PLXD1	37.34	3.15	Q9Y4D7-2
Q71DI3|H32	85.25	3.03	Q71DI3
P16401|H15	135.75	2.9	P16401
B3KUP2|B3KUP2	108.93	2.82	B3KUP2
P04040|CATA	72.61	2.79	P04040
P22102|PUR2	103.29	2.74	P22102
P42892-2|ECE1	38.96	2.72	P42892-2

P41221|WNT5A	14.7	2.72	P41221
B4E1M5|B4E1M5	67.34	2.64	B4E1M5
P23381|SYWC	57.57	2.63	P23381
O60343-2|TBCD4	59.48	2.6	O60343-2
E5RFF9|E5RFF9	19.58	2.6	E5RFF9
B7Z576|B7Z576	29.49	2.59	B7Z576
E7ET98|E7ET98	56.63	2.49	E7ET98
F5GWE6|F5GWE6	44.01	2.49	F5GWE6
F5GZ28|F5GZ28	41.29	2.48	F5GZ28
P04181|OAT	153.35	2.47	P04181
E7EQG2|E7EQG2	111.66	2.45	E7EQG2
Q92820|GGH	39.85	2.45	Q92820
P00491|PNPH	31.44	2.44	P00491
P60842|IF4A1	292.06	2.38	P60842
P41091|IF2G	168.16	2.3	P41091
B2RDM2|B2RDM2	117.68	2.29	B2RDM2
P67936-2|TPM4	307.35	2.28	P67936-2
Q5T4L4|Q5T4L4	44.69	2.28	Q5T4L4
P07741|APT	71.56	2.27	P07741
O14950|ML12B	52.37	2.25	O14950
P84243|H33	127.5	2.13	P84243
P08473|NEP	32.44	2.13	P08473
Q5VTU3|Q5VTU3	69.28	2.12	Q5VTU3
A6QRJ1|A6QRJ1	32.26	2.12	A6QRJ1
P61201-2|CSN2	41.11	2.1	P61201-2
F8WJN3|F8WJN3	98.76	2.07	F8WJN3
P15374|UCHL3	96.84	2.07	P15374
Q96EK5|KBP	45.05	2.07	Q96EK5
Q96JD6|AKCL2	46.56	2.04	Q96JD6
F8VXB4|F8VXB4	607.74	2.01	F8VXB4
E7EM48|E7EM48	323.76	2.01	E7EM48
P08648|ITA5	67.96	2.01	P08648
B4DFL2|B4DFL2	259.91	1.98	B4DFL2
Q8WXX5|DNJC9	42.81	1.93	Q8WXX5
E9PB21|E9PB21	24.47	1.93	E9PB21
A6NHK2|A6NHK2	13.74	1.93	A6NHK2
P13010|XRCC5	109.9	1.92	P13010
F8VTL3|F8VTL3	474.85	1.91	F8VTL3
B3SXQ8|B3SXQ8	296.74	1.9	B3SXQ8
F5H335|F5H335	193.35	1.9	F5H335
P11766|ADHX	188.46	1.9	P11766
Q13177|PAK2	104.25	1.89	Q13177
Q13526|PIN1	82.5	1.88	Q13526
C9J5P2|C9J5P2	248.23	1.87	C9J5P2

Q5T9B7|Q5T9B7	52.71	1.87	Q5T9B7
B7Z4B8|B7Z4B8	71.63	1.84	B7Z4B8
F6RPD3|F6RPD3	41.87	1.84	F6RPD3
F8WE04|F8WE04	278.21	1.83	F8WE04
Q9Y281|COF2	146.01	1.83	Q9Y281
Q63ZY3-2|KANK2	95.87	1.82	Q63ZY3-2
F5CTF3|F5CTF3	25.16	1.82	F5CTF3
P23284|PPIB	207.26	1.81	P23284
Q4TTY5|Q4TTY5	54.37	1.81	Q4TTY5
O60271-7|JIP4	62.16	1.79	O60271-7
Q99741|CDC6	38.13	1.79	Q99741
Q9Y3C4-3|TPRKB	29.21	1.79	Q9Y3C4-3
B4DY81|B4DY81	21.83	1.79	B4DY81
Q15287|RNPS1	69.46	1.78	Q15287
P09486|SPARC	20.86	1.78	P09486
B7Z7L3|B7Z7L3	75.84	1.75	B7Z7L3

Proteins down-regulated in RPE1 E7 expressing cells

Accession	Score	Fold	UniProtKB ID
P62495|ERF1	56.18	136.12	P62495
D9YZV2|D9YZV2	187.8	110.74	D9YZV2
P02533|K1C14	75.49	55.81	P02533
B5MC82|B5MC82	68.39	47.6	B5MC82
E9PQN9|E9PQN9	73.66	44.5	E9PQN9
Q9UNX4|WDR3	25.74	38.28	Q9UNX4
Q9BPX5|ARP5L	67.12	25.33	Q9BPX5
B3KQ95|B3KQ95	34.55	24.25	B3KQ95
F8WCG3|F8WCG3	67.96	20.12	F8WCG3
F8VY02|F8VY02	118.36	19.77	F8VY02
F5H6X2|F5H6X2	57.06	16.15	F5H6X2
P35527|K1C9	40.88	12.99	P35527
F5H2F4|F5H2F4	138.84	10.85	F5H2F4
E9PAV3|E9PAV3	135.16	10.12	E9PAV3
Q9H3N1|TMX1	49.87	9.51	Q9H3N1
F8W7C6|F8W7C6	214.25	7.07	F8W7C6
Q8N995|Q8N995	53.99	6.26	Q8N995
E9PS42|E9PS42	28.74	3.58	E9PS42
Q92520|FAM3C	23.59	2.88	Q92520
O94925-3|GLSK	50.78	2.67	O94925-3
Q9Y3D6|FIS1	43.54	2.63	Q9Y3D6
F5GXL9|F5GXL9	72.11	2.6	F5GXL9
F8WAM4|F8WAM4	99.29	2.57	F8WAM4
P17931|LEG3	39.16	2.57	P17931

P31689|DNJA1	70.02	2.54	P31689
F5H2A4|F5H2A4	18.29	2.5	F5H2A4
P12270|TPR	155.25	2.49	P12270
Q16222-3|UAP1	147.35	2.46	Q16222-3
P00750-3|TPA	18.61	2.43	P00750-3
P13645|K1C10	304.83	2.36	P13645
P20618|PSB1	80.7	2.31	P20618
P00441|SODC	48.18	2.31	P00441
F5GZI1|F5GZI1	61.87	2.3	F5GZI1
Q9Y5Z4-2|HEBP2	26.19	2.3	Q9Y5Z4-2
Q13242|SRSF9	53.97	2.28	Q13242
P04264|K2C1	744.72	2.25	P04264
E9PP17|E9PP17	22.08	2.23	E9PP17
B4E2W0|B4E2W0	25.51	2.2	B4E2W0
Q86VZ5|SMS1	16.94	2.2	Q86VZ5
B2RCS5|B2RCS5	92.91	2.17	B2RCS5
P53675-2|CLH2	332.88	2.12	P53675-2
C9JP00|C9JP00	38.92	2.12	C9JP00
A8MVZ9|A8MVZ9	118.95	2.1	A8MVZ9
P14868|SYDC	266.02	2.02	P14868
B4DHY1|B4DHY1	127.31	2.02	B4DHY1
P38646|GRP75	98.01	2.02	P38646
Q9Y678|COPG	76.32	1.99	Q9Y678
Q06210-2|GFPT1	57.74	1.96	Q06210-2
B4E040|B4E040	34.66	1.96	B4E040
F5H740|F5H740	117.2	1.95	F5H740
A8MSH7|A8MSH7	70.67	1.94	A8MSH7
E7ERJ4|E7ERJ4	167.68	1.92	E7ERJ4
P57103-2|NAC3	34.79	1.92	P57103-2
D6RER5|D6RER5	64.6	1.91	D6RER5
B4DXJ9|B4DXJ9	187.91	1.9	B4DXJ9
C9JYJ0|C9JYJ0	42.55	1.9	C9JYJ0
D6R9W4|D6R9W4	172.8	1.88	D6R9W4
P40222|TXLNA	56.84	1.85	P40222
E9PMP7|E9PMP7	321.76	1.84	E9PMP7
Q99536|VAT1	92.22	1.83	Q99536
P43034|LIS1	66.88	1.83	P43034
E7EWH4|E7EWH4	86.3	1.82	E7EWH4
P62244|RS15A	78.1	1.82	P62244
Q12931|TRAP1	43.18	1.82	Q12931
B4DNU6|B4DNU6	35.34	1.82	B4DNU6
P49913|CAMP	13.95	1.82	P49913
Q01813|K6PP	438.92	1.81	Q01813
Q08499-6|PDE4D	27.07	1.81	Q08499-6

P62805|H4	304.52	1.8	P62805
B5ME19|B5ME19	182.11	1.8	B5ME19
O15144|ARPC2	94.01	1.8	O15144
O15042-3|SR140	81.86	1.8	O15042-3
E9PJF7|E9PJF7	92.89	1.79	E9PJF7
P17858|K6PL	136.69	1.78	P17858
P13726|TF	131.49	1.78	P13726
F2Z388|F2Z388	94.93	1.78	F2Z388
B4DEG8|B4DEG8	66.15	1.78	B4DEG8
F5H307|F5H307	25.56	1.78	F5H307
P61221|ABCE1	129.65	1.77	P61221
O14828-2|SCAM3	23.06	1.76	O14828-2
P07384|CAN1	83.18	1.75	P07384
D6QS48|D6QS48	79.45	1.75	D6QS48
Q96FQ6|S10AG	51.83	1.75	Q96FQ6

Supplementary Figure 1:

Presentation of original immunoblots. The cropped parts of immunoblots were indicated with boxes.

Figure 2a

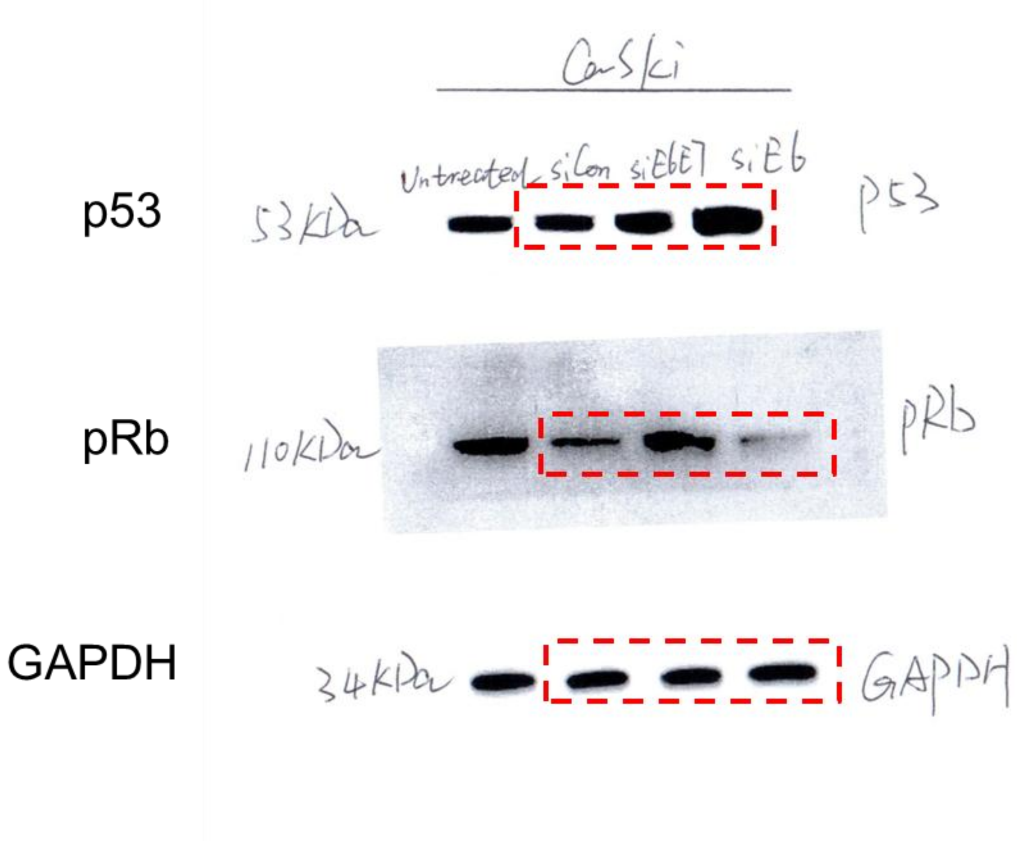

Supplementary Figure 2:

Presentation of original immunoblots.

Figure 3a

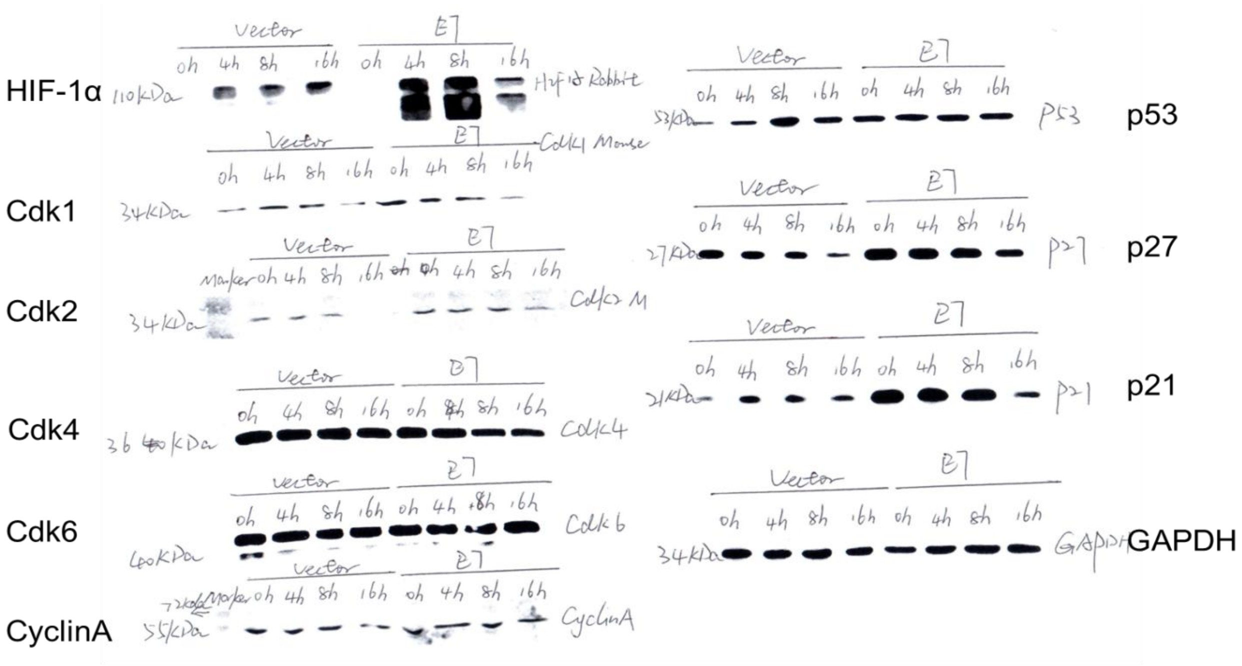

Figure 3b

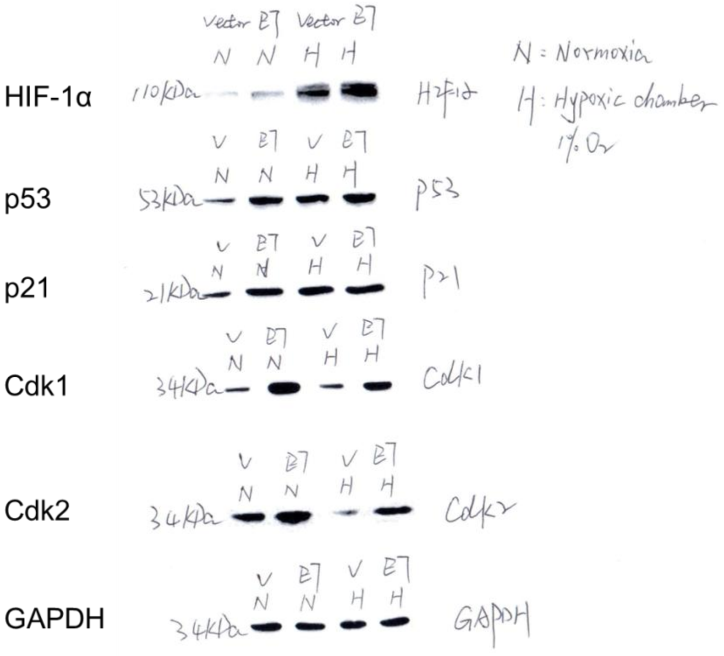

Supplementary Figure 3:

Presentation of original immunoblots.

Figure 4c

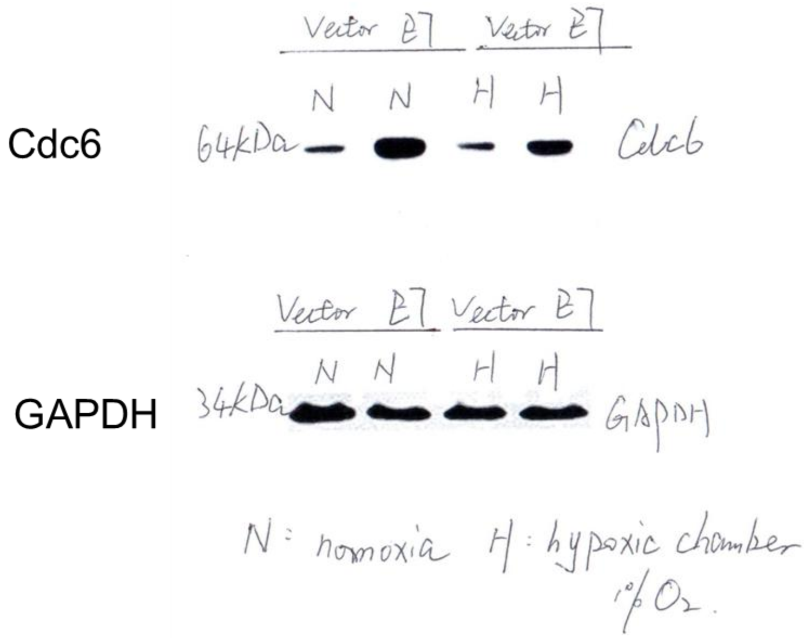

Figure 5a

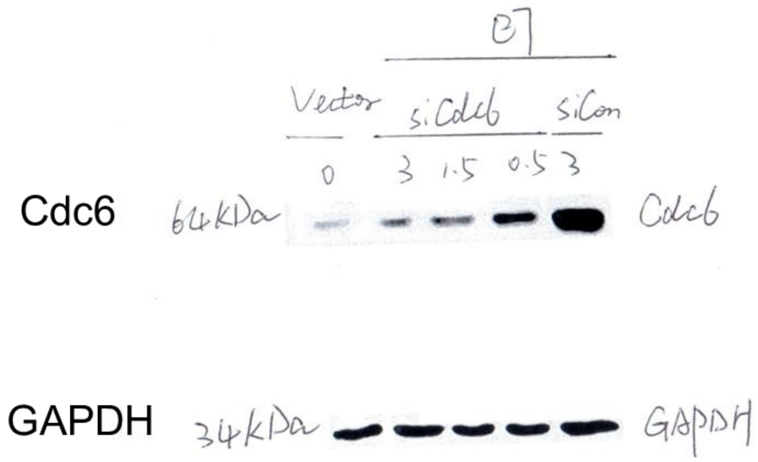

Supplement: Supplementary file 1 — Supplementary Information [file 41598_2017_3060_MOESM1_ESM.pdf]
